# Prenatal and Early Childhood Exposure to Lead and Repeated Measures of Metabolic Syndrome Risk Indicators From Childhood to Preadolescence

**DOI:** 10.3389/fped.2021.750316

**Published:** 2021-10-29

**Authors:** Karla Muciño-Sandoval, Ana Carolina Ariza, Eduardo Ortiz-Panozo, María Luisa Pizano-Zárate, Adriana Mercado-García, Robert Wright, Martha Maria Téllez-Rojo, Alison P. Sanders, Marcela Tamayo-Ortiz

**Affiliations:** ^1^Research Center for Health and Nutrition, National Institute of Public Health, Cuernavaca, Mexico; ^2^Research Center for Population Health, National Institute of Public Health, Cuernavaca, Mexico; ^3^Division for Research and Community Interventions, National Institute of Perinatology, Mexico City, Mexico; ^4^Departments of Environmental Medicine and Public Health and Pediatrics, Icahn School of Medicine at Mount Sinai, New York, NY, United States; ^5^Occupational Health Research Unit, Mexican Institute of Social Security (IMSS), Mexico City, Mexico

**Keywords:** lead, prenatal exposure, metabolic syndrome, early childhood, heavy metals

## Abstract

**Background:** Exposure to lead (Pb) during the early life stages has been associated with the development of metabolic syndrome (MetS). Longitudinal studies of Pb exposure in critical developmental windows in children are limited.

**Methods:** Our study included 601 mother–child dyads from the PROGRESS (Programming Research in Obesity, Growth, Environment and Social Stressors) birth cohort. Blood lead levels (BLLs) were assessed during the second and third gestational trimesters, in cord blood at delivery, and at ages 1, 2, and 4 years. Bone lead levels in the patella and tibia were assessed at 1 month postpartum and evaluated in separate models. To account for cumulative exposure (prenatal, postnatal, and cumulative), we dichotomized the BLLs at each stage visit and determined the following: “higher” if a BLL was at least once above the median (HPb) and “lower” if all BLLs were below the median (LPb). We analyzed fasting glucose, HbA1c, triglycerides (TGs), total cholesterol (TC), high-density lipoprotein cholesterol (cHDL), low-density lipoprotein cholesterol (cLDL), body mass index, waist circumference (WC), body fat percentage, and systolic (SBP) and diastolic blood pressure (DBP) at two study visits between 6 and 12 years of age and created cutoff points based on the clinical guidelines for each indicator. Mixed effects models were used to analyze each outcome longitudinally for each BLL score, adjusting for child's sex, size for gestational age, child's age, maternal parity, mother's age, and socioeconomic status.

**Results:** We observed associations for HPb exposure and TC in all stages (OR = 0.53, 95%CI = 0.32–0.86) and postnatally (OR = 0.59, 95%CI = 0.36–0.94) and for prenatal HPb and TGs (OR = 0.65, 95%CI = 0.44–0.95). HPb at all stages was associated with WC (OR = 0.27, 95%CI = 0.08–0.86), BMI (OR = 0.33, 95%CI = 0.11–0.99), SBP (OR = 0.53, 95%CI = 0.32–0.85), and DBP (OR = 0.57, 95%CI = 0.34–0.95). Pb levels in the patella were associated with cHDL (OR = 1.03, 95%CI = 1.00–1.07) and those in the tibia with TGs (OR = 0.95, 95%CI = 0.91–0.99).

**Conclusion:** Early life exposure to Pb may alter early indicators of MetS. A follow-up of these children will allow for more definition on the impact of longer-term exposures.

## Introduction

Exposure to lead (Pb) has been declared second on the list of 10 highest priority toxic substances to public health due to established multisystem toxicity and widespread exposure by the World Health Organization (WHO) and the Agency for Toxic Substances and Disease Registration (ATSDR) (1, 2).

During pregnancy, Pb has potential impacts on fetal health due to its ability to cross the placental barrier (3); additionally, endogenous Pb exposure increases due to bone resorption (4, 5). Postnatally, Pb can also be transferred to the newborn through breast milk (3, 6, 7). Developmental windows, including *in utero* and early childhood, are key to study the effects of Pb exposure on organ growth and development, which may contribute to adverse health outcomes later in life (7).

In Mexico, the principal route of exposure to Pb is gastrointestinal, *via* the consumption of food prepared, served, or stored in lead-glazed low-temperature ceramics as lead will leach into food, especially with acidic food frequent in Mexican cuisine (1, 3, 8). Once inside the human body, Pb can traverse to the brain, heart, liver, and kidneys, altering their normal biological functions (9). One of the mechanisms of Pb toxicity is the production of reactive oxygen species, resulting in various adverse health effects such as oxidative damage to DNA, proteins, and lipids and increased lipid peroxidation in the cell membrane (9, 10). In the cardiovascular system, Pb is capable of displacing divalent cations, particularly Ca^2+^, affecting the ion channels and other receptor functions in the brain, such as the *N*-methyl-d-aspartate receptor. In the cardiovascular system, it might modify the permeability of blood vessels, leading to vascular damage, cardiac toxicity, cardiac dysfunction, and hypertension. Another mechanism of action is its role as an endocrine disruptor, functioning as endogenous hormones and mimicking endocrine effects (4, 11). Moreover, results from *in vitro* and animal models suggest that Pb can modify the differentiation of progenitor cells, increase adipogenesis, and alter glucose homeostasis, accounting for some of the observed comorbidities present in metabolic syndrome (MetS) (2, 12).

MetS is defined as a set of physiological, biochemical, clinical, and metabolic factors that increase the risk of cardiovascular diseases such as myocardial infarction, atherosclerosis, systemic arterial hypertension, cerebrovascular disease, and diabetes mellitus type 2 (13, 14). The International Diabetes Federation (IDF) defines the diagnostic criteria for MetS in the adult population as the presence of at least three out of five factors: abdominal obesity, elevated triglycerides, low levels of high-density lipoprotein cholesterol, high blood pressure, and altered fasting glucose (2, 15). However, there is no consensus on the applicability of these cutoff points in children and adolescents (13, 14, 16). According to the IDF definition, MetS should not be formally diagnosed in children under the age of 10. However, there is evidence that the abdominal circumference, body mass index, blood pressure, lipoprotein blood levels, and blood glucose can be altered beginning in early childhood and that these profiles may track into adolescence and adulthood (13, 14, 17).

The presence of MetS during childhood and preadolescence significantly increases the risk of diabetes mellitus type 2 and cardiovascular disease in adulthood (18, 19). The prevalence of MetS can be attributed to factors such as poor diet, sedentary lifestyle, and social or genetic factors (20). Intriguingly, there is growing evidence supporting a link between Pb exposure and the development of MetS (21) and various pathologies (17, 20, 22). For example, exposure to Pb during pregnancy and lactation, in animal models, was associated with the development of insulin resistance and obesity, and this association was greater among males than females (18). However, there is conflicting evidence of Pb exposure during the prenatal stage and the subsequent levels of cholesterol (total, high density, and low density) in pediatric cohorts (17, 20, 21). Some studies in children have reported sexually dimorphic sensitivity between the effect of exposure to Pb and the presence of different indicators of MetS (20, 21).

Despite growing evidence supporting a link between Pb exposure and MetS risk factors, there are only a few existing longitudinal studies among pediatric populations that examined perinatal Pb exposure and its relationship with increased risk of MetS in childhood and preadolescence (17, 18). The aim of this study was to explore the association between prenatal and early childhood exposure to Pb and repeated measures of MetS risk indicators in children between 6 and 12 years of age.

## Materials and Methods

### Study Population

The participants of this study are part of the PROGRESS (Programming Research in Obesity, Growth, Environment and Social Stressors) prenatal cohort from Mexico City. Pregnant women receiving medical and prenatal care in the Mexican Social Security Institute (IMSS) were invited to participate. The recruitment period took place between July 2007 and February 2011. To be eligible for the study, women had to be at <20 weeks gestation, ≥18 years of age, without current or prior pathologies of heart or kidney disease, accessibility to a phone, plan to reside in Mexico City for the next 3 years after their admission to the study, not use steroid drugs (including glucocorticoids) or antiepileptic medications, and not consume alcohol daily. The institutional boards and ethics committees of Harvard School of Public Health, Icahn School of Medicine in Mount Sinai, and the National Institute of Public Health in Mexico approved the project, as well as the collaborating institutions: National Institute of Perinatology (INPer), IMSS, and the American British Cowdray (ABC) Medical Center. Participants granted their informed consent for participation in the study. There were 948 women followed during pregnancy and who gave birth to a live child; children had follow-up visits at 1, 6, 12, 18, 24, 48, and 72, and 96 months of age. The cohort had most of its dropouts between birth and at the stage 24 visit and has maintained stable retention at around *n* = 600 mother–child dyads since.

The inclusion criteria for this study were: having data of at least one blood lead level (BLL) during pregnancy and one postpartum (i.e., second and third trimesters of pregnancy, birth, and at 12, 24, and 48 months of age) and outcome data (i.e. MetS indicators) at 72 and 96 months. Some of the children's blood samples from stages 12 and 24 were randomly lost during transportation from Mexico to the laboratory in the USA, leaving a total of *n* = 174 and *n* = 247, respectively. For stage 48, we have the data for 501 children. Children who had an extremely low birth weight equivalent to <1,500 g and gestational age <32 weeks were excluded (*n* = 21). On average, 601 children had data for at least one incidence of MetS for stage 72 and 540 had data for stage 96, with varying covariate data during the study follow-up that are indicated in the corresponding results table.

### Pb Exposure

Prenatal exposure was evaluated with blood measurements from women in the second and third trimesters of pregnancy and in umbilical cord blood. Postnatal Pb exposure was assessed from children's blood samples collected at 12, 24, and 48 months of age. All blood samples were drawn in trace metal-free tubes and refrigerated at 4°C until shipment to the laboratory where they were frozen at −20°C until analysis. Pb concentration was measured by external calibration using the Agilent 8800 ICP Triple Quad (Agilent, Santa Clara, CA, USA) in MS/MS mode in the Trace Metals Laboratory at the Icahn School of Medicine at Mount Sinai. The limit of detection was <0.2 μg/dl and the instrument precision (given as %RSD) was ~5%. Good precision and accuracy were shown using blinded quality control samples obtained from the Maternal and Child Health Bureau and the Wisconsin State Laboratory of Hygiene Cooperative Blood Lead Proficiency Testing Program. BLLs were analyzed as continuous variables (given in micrograms per deciliter) and as categorical variables classified according to the median exposure per stage, described below.

To account for cumulative exposure, at the prenatal, postnatal, and cumulative stages, we dichotomized the BLLs using the median for each study visit as the cutoff point. Two categories were obtained: “higher,” when a BLL was at least one time above the median (HPb), and “lower,” if all BLLs were below the median (LPb).

We measured bone Pb levels at 1 month postpartum in maternal tibia (mid-tibial shaft, cortical bone) and patella (trabecular bone) using a K-shell X-ray fluorescence instrument for 30 min in each leg. The measures were computed, averaged, and weighted by the inverse of the proportion of the measurement error corresponding to each determination. Negative values, produced when the true values are below the detection limit of the instrument, were imputed with random draws from a uniform distribution between 0 and the lower limit of 2 μg lead/g bone mineral (23).

### Risk Factor Indicators of MetS

Metabolic syndrome risk factors (IMetS), which were assessed at the 72- and 96-month visits, included the following: glucose, glycated hemoglobin (HbA1c), total cholesterol (TC), triglycerides (TGs), high-density lipoprotein cholesterol (cHDL), low-density lipoprotein cholesterol (cLDL), body fat percentage (BF%), waist circumference (WC), body mass index (BMI), systolic blood pressure (SPB), and diastolic blood pressure (DBP).

Fasting venous blood was drawn to obtain blood levels of TC, cHDL, cLDL, TGs, glucose, and HbA1c. Enzyme methods (Roche Diagnostics, Indianapolis, IN, USA) were used to obtain measures of TC, cHDL, cLDL, and TGs. The levels of HbA1c were determined using the Miura 200 automated analyzer (ISE S.r.l., Rome, Italy). The weight, height, and waist circumference were measured during both follow-ups in triplicate and the average of each measurement obtained. The weight and height data were used to calculate the BMI (weight/size^2^) and the *z*-score with BMI for age indicator using the WHO “Anthro” software (24). Total adipose tissue was measured using the InBody 370 or 230 tetrapolar bioelectric impedance equipment (Biospace Co., Ltd., Los Angeles, CA, USA). BF% was obtained by estimating the total adipose tissue divided by the total body mass. WC was measured at the midpoint between the last floating rib and the iliac crest. Standardized personnel performed all anthropometric measurements.

SBP and DBP were measured at rest using an automated Spacelabs Healthcare monitor, Ultralite Ambulatory 90217 (Snoqualmie, WA, USA). This measurement was taken in duplicate with a 3-min difference between each, and the average of the measurements was used.

IMetS were categorized according to the different cutoff points (24, 25) considering high levels in blood if: glucose was ≥100 mg/dl, HbA1c was ≥5.7%, TC was ≥170 mg/dl, cHDL was <45 mg/dl, cLDL was >110 mg/dl, and TGs were >75 mg/dl for children 9 years of age or younger and >90 mg/dl for children 10 years of age or older. The cutoff point for BF% and WC was the 80th percentile. BMI *z*-scores were classified according to the WHO Child Growth Standards (24): underweight, less than −2 SD; normal weight, greater than −2 to less than +1 SD; overweight, greater than +1 SD; and obese, greater than +2 SD. The cutoff point for SPB and DBP was the 90th percentile.

### Covariates

The following variables were considered for model adjustment: maternal age at recruitment, socioeconomic status (SES), parity collected through standardized questionnaires during the second trimester of pregnancy, child's size for gestational age (Fenton score), sex, and age at the 72- and 96-month visits. SES was measured using the Mexican Association of Market Intelligence and Opinion Agencies questionnaire. With reference to our study population, which belongs to a middle–lower SES, we collapsed the variable into three categories: lower, medium, and higher. Parity considered two categories (including the current pregnancy): primiparous (less than or one pregnancy) and two or more pregnancies.

### Statistical Analysis

Descriptive analyses were carried out for the evaluation of extreme or implausible values in the database. In the case of continuous variables, the mean and standard deviations were obtained, as well as the ranges or the median and interquartile range, as appropriate. Frequencies and percentages were obtained for categorical variables. Student's *t*-test and the Mann–Whitney *U* test were used for continuous variables and the chi-square test used for categorical variables.

To assess the association between exposure to Pb and IMetS, linear mixed effects models and logistic mixed effects models were utilized. BLLs were evaluated both as continuous and dichotomous. IMetS were assessed as continuous and categorized. The models were adjusted for sex, maternal and child ages, parity, SES, and the Fenton score. Secondary analyses were additionally adjusted for the BMI *z*-scores to ensure that obesity did not impact the results. Models were utilized considering three developmental windows: prenatal, postnatal, and cumulative. We stratified our models by sex to assess possible effect modifications of the associations. Outliers of the IMetS were excluded (*n* = 4), three with biologically implausible BF% data and one with a BMI *z*-score for age ≥5 SD. All analyses were performed using Stata version 14 software (StataCorp LLC, College Station, TX, USA).

As a sensitivity analysis, mixed effects models were utilized with a subsample that had the complete follow-up data to verify that loss to follow-up and the presence of missing values did not affect the results.

## Results

General characteristics of the 601 mother–child dyads included in this study are described in Table 1. Women's mean age at enrollment was 27.1 ± 5.5 years. More than half of the participants had two or more previous pregnancies (62%), and most had lower SES. At birth, children were an average of 3 ± 0.5 kg in weight, with a gestational age of 38.1 weeks. There were 48 children with low birth weight and 39 who were born preterm. The median BLLs were higher in the prenatal stages compared to those in the postnatal stages.

**Table 1:** Basal characteristics in mother–child dyads from the PROGRESS cohort.

**Characteristics**	**Mean (SD) or median (Q1–Q3)**		
	**Total (*N* = 601)**	**Boys (*n* = 308)**	**Girls (*n* = 293)**
**Maternal characteristics**			
Maternal age (years)^a^	27.1 (5.5)	27.3 (5.4)	26.9 (5.7)
Socioeconomic status^b^			
Lower	52.6%	52.9%	52.2%
Medium	37.3%	36.7%	37.9%
Higher	10.1%	10.4%	9.9%
Parity (2)			
Primiparous	38.4%	36.4%	40.6%
Multiparous	61.6%	63.6%	59.4%
**Children's characteristics**			
Birth weight (kg)^*^	3.1 (0.4)	3.1 (0.4)	3.0 (0.4)
Gestational age (weeks)^c^	38.8 (1.5)	38.8 (1.6)	38.8 (1.5)
BLLs in the prenatal stage			
Second trimester (μg/dl)^c^^*^	2.9 (1.9–4.4)	3.1 (2.0–4.6)	2.7 (1.9–4.2)
Third trimester (μg/dl)^c^	3.1 (2.0–4.8)	3.1 (2.0–4.9)	3.0 (1.9–4.6)
At birth in umbilical cord (μg/dl)^c^	2.2 (1.4–3.7)	2.4 (1.4–3.7)	2.1 (1.4–3.8)
Bone Pb levels: patella	3.4 (1.3–8.9)	3.0 (1.2–8.8)	4.3 (1.4–9.5)
Bone Pb levels: tibia	3.0 (1.1–7.5)	3.1 (1.4–7.4)	2.9 (0.9–7.6)
BLLs in the postnatal stage^d^			
1 year (μg/dl)^c^	2.0 (1.6–2.9)	2.1 (1.5–3.2)	2.0 (1.6–2.6)
2 years (μg/dl)^c^	2.2 (1.6–3.1)	2.4 (1.7–3.2)	2.0 (1.5–3.0)
4 years (μg/dl)^c^	1.7 (1.3–2.5)	1.7 (1.3–2.5)	1.7 (1.3–2.6)

*BLLs, blood lead levels*.

* *p < 0.05 for the difference between sex categories (Student's t-test or Mann–Whitney U test for numerical variables and Pearson's test of independence for categorical variables)*.

a *With values shown as mean (SD)*.

b *Categorical variables with values shown as frequency (%)*.

c *With values shown as median and interquartile ranges*.

d *BLLs at 1 year: N = 139, n = 67 boys and n = 72 girls; at 2 years: N = 203, n = 99 boys and n = 104 girls; at 4 years: N = 475, n = 242 boys and n = 233 girls*.

The characteristics of the 601 participants included in these analyses were compared to those of non-participants, and the differences were not statically significant (results not shown).

Table 2 shows the descriptive characteristics of the IMetS for boys and girls at the 72- and 96-month study visits. Most indicators showed statistically significant differences between visits, and we observed a considerable increase in the prevalence of overweight and obesity. Boys had higher glucose and lower triglycerides than did girls at the 96-month study visit (*p* < 0.05).

**Table 2:** Children's metabolic syndrome risk factors by sex and study visit.

**Indicators**	**Boys**			**Girls**		
	**Median**		***p-value***	**Median**		***p-value***
	**Stage 72**	**Stage 96**		**Stage 72**	**Stage 96**	
Glucose (mg/dl)^**^	88.6	87.1	0.157	85.7	85.3	0.822
HbA1C (%)	5.4	4.9	<0.001	5.4	5.1	<0.001
TC (mg/dl)	161.0^*^	154	0.092	166^*^	157	<0.001
TGs (mg/dl)^**^	68.0	75.0	<0.001	77.5	86.0	<0.001
cHDL (mg/dl)	50.9	51.1	0.064	49.4	46.7	<0.001
cLDL (mg/dl)	92.8^*^	87.7	<0.01	97.1^*^	89.9	<0.001
Body fat (%)	22.6^*^	29.6	<0.001	24.4^*^	30.5	<0.001
WC (cm)	54.3	65.6	<0.001	54.9	65.1	<0.001
BMI (kg/m^2^)	15.7	18.1	<0.001	15.8	18.0	<0.001
Systolic blood pressure (mmHg)	102.0^*^	112.3	<0.001	100.5^*^	110.6	<0.001
Diastolic blood pressure (mmHg)	61.5	70.7	<0.001	60.5	69.5	<0.001

*For stage 72, glucose, TC, TGs, cHDL, and cLDL: n = 265 boys and n = 260 girls; WC and BMI: n = 308 boys and n = 293 girls; systolic and diastolic blood pressure: n = 286 boys and n = 272 girls; HbA1c: n = 264 boys and n = 261 girls; BF%: n = 277 boys and n = 269 girls*.

*For stage 96, glucose, TC, TGs, cHDL, and cLDL: n = 259 boys and n = 250 girls; WC and BMI: n = 275 boys and n = 265 girls; systolic and diastolic blood pressure: n = 265 boys and n = 253 girls; HbA1c: n = 257 boys and n = 251 girls; BF%: n = 274 boys and n = 264 girls. The p-values shown represent the differences between stages*.

*TC, total cholesterol; TGs, triglycerides; cHDL, high-density lipoprotein cholesterol; cLDL, low-density lipoprotein cholesterol; WC, waist circumference; BF%*.

* *p < 0.05 for differences between sex at stage 72 (Mann–Whitney U test for numerical variables)*;

** *p < 0.05 for differences between sex at stage 96 (Mann–Whitney U test for numerical variables)*.

The main finding for IMetS classified according to the cutoff points was the presence of at least one IMetS in children: 61.6% for the stage 72 study visit and 63.7% for the stage 96 study visit. The prevalence of glucose ≥100 mg/dl was found to be higher in boys than that in girls. Most of the IMetS showed an increase in prevalence between stages 72 and 96 and were more prevalent among overweight and obese participants. Between stages 72 and 96, increases of 6 percentage points for overweight and 13.6 percentage points for obesity were found in boys. Similar increases were found in girls, 8.6 percentage points for overweight and 7.5 percentage points for obesity (Table 3).

**Table 3:** Children's metabolic syndrome risk factors according to the cutoff points by sex and study visit.

**Indicators**	**Boys**		**Girls**	
	**Stage 72**	**Stage 96**	**Stage 72**	**Stage 96**
	***n* (%)**	***n* (%)**	***n* (%)**	***n* (%)**
Glucose, ≥100 mg/dl	25 (9.4)	26 (10.1)	18 (6.9)	15 (6.0)
HbA1C, ≥5.7%	32 (12.5)	17 (6.6)	27 (10.8)	23 (9.2)
Total cholesterol, >170 mg/dl	65 (24.9)	78 (30.6)	98 (38.7)	76 (30.6)
Triglycerides, >75 or >90 mg/dl	104 (39.7)	96 (37.9)	134 (51.7)	120 (48.6)
cHDL, <45 mg/dl	74 (27.9)	78 (30.1)	83 (31.9)	109 (43.6)
cLDL, >110 mg/dl	36 (13.6)	44 (17.0)	58 (22.3)	48 (19.3)
Body fat percentage, >80th percentile	27 (9.7)	27 (9.8)	27 (10.0)	26 (9.8)
Waist circumference, >80th percentile	61 (19.8)	55 (20.0)	58 (19.8)	53 (20.0)
BMI for age, *z*-scores				
Underweight, less than−2 SD	7 (2.3)	4 (1.4)	1 (0.3)	3 (1.1)
Normal, greater than −2 to less than +1 SD	214 (69.7)	140 (50.9)	212 (72.4)	147 (55.5)
Overweight, greater than +1 SD	46 (15.0)	58 (21.1)	50 (17.1)	68 (25.7)
Obesity, greater than +2 SD	40 (13.0)	73 (26.6)	30 (10.2)	47 (17.7)
Systolic blood pressure, >90th percentile	27 (9.4)	25 (9.4)	26 (9.6)	23 (9.1)
Diastolic blood pressure, >90th percentile	27 (9.4)	26 (9.8)	27 (9.9)	25 (9.9)

*cHDL, high-density lipoprotein cholesterol; cLDL, low-density lipoprotein cholesterol*.

Table 4 shows the results of the BLL mixed effects logistic models for prenatal, postnatal, and all stages (i.e., IMetS categorized according to the cutoff points). According to the results, we found that children with HPb in the prenatal stage were 47% (OR = 0.53, 95%CI = 0.31–0.99) less likely to have TC > 170 mg/dl compared to children with LPb. Similar results were observed for postnatal BLLs (OR = 0.59, 95%CI = 0.36–0.94). We observed an association between prenatal HPb and TGs (OR = 0.65, 95%CI = 0.44–0.95). Children with postnatal HPb were 73% (OR = 0.27, 95%CI = 0.08–0.86) less likely to have a WC above the 80th percentile. For the “all stages” model, children with HPb were less likely to have SBP (OR = 0.53, 95%CI = 0.32–0.85) and DBP (OR = 0.57, 95%CI = 0.34–0.95) above the 80th percentile. We also observed this association in the prenatal model (OR = 0.60, 95%CI = 0.37–0.98).

**Table 4:** Association of higher blood lead exposure levels (above the median) and indicators of metabolic syndrome categorized according to the cutoff points.

**Indicators**	**All stages^a^**		**Prenatal^a^**		**Postnatal^a^**	
	**OR**	**95% CI**	**OR**	**95% CI**	**OR**	**95% CI**
Glucose, ≥100 mg/dl	1.22	0.64–2.33	0.79	0.46–1.37	0.97	0.56–1.68
HbA1c, ≥5.7%	0.87	0.50–1.48	0.90	0.55–1.44	1.47	0.85–2.52
Total cholesterol, >170 mg/dl	**0.53**	**0.32−0.86**	*0.67*	*0.43*–*1.02*	**0.59**	**0.36–0.94**
Triglycerides, >75 or >90 mg/dl	0.72	0.46–1.11	**0.65**	**0.44–0.95**	*0.69*	*0.46*–*1.04*
cHDL, <45 mg/dl	1.46	0.88–2.44	1.37	0.88–2.12	1.08	0.66–1.75
cLDL, >110 mg/dl	0.75	0.42–1.33	0.81	0.48–1.34	0.93	0.53–1.61
Body fat percentage, >80th percentile	0.78	0.20–2.97	1.62	0.53–4.93	0.65	0.20–2.05
Waist Circumference, >80th percentile	**0.27** ^b^	**0.08–0.86**	0.77	0.29–1.99	*0.37*	*0.13*–*1.07*
BMI for age, *z*-scores	**0.33**	**0.11–0.99**	*0.43*	*0.16*–*1.14*	0.60	0.20–1.68
Systolic blood pressure, >90th percentile	**0.53**	**0.32–0.85**	*0.66*	*0.41*–*1.02*	0.76	0.46–1.24
Diastolic blood pressure, >90th percentile	**0.57**	**0.34–0.95**	**0.60**	**0.37–0.98**	0.76	0.45–1.26

*cHDL, high-density lipoprotein cholesterol; cLDL, low-density lipoprotein cholesterol; TC, total cholesterol; WC, waist circumference*.

a *Models adjusted for maternal characteristics (socioeconomic status, maternal age, and parity) and characteristics of infants (sex, size for gestational age, and infant age)*.

b *Results in bold with statistically significant differences (p < 0.05)*.

*^c^Results in italics with marginally significant differences (p < 0.09)*.

Body fat percentage models only adjusted for maternal and infant ages because of the limited number of each category in categorical covariates. The glucose model in the postnatal stage was adjusted for socioeconomic status, sex, maternal age, infant age, and parity. Glucose, TC, cHDL, and cLDL: *n* = 586 for all stages, *n* = 585 for the prenatal stage, and *n* = 509 for the postnatal stage. Glycosylated hemoglobin and TGs: *n* = 583 for all stages, *n* = 582 for the prenatal stage, and *n* = 508 for the postnatal stage. WC and BMI: *n* = 601 for all stages, *n* = 600 for the prenatal stage, and *n* = 519 for the postnatal stage. Systolic and diastolic blood pressure: *n* = 599 for all stages, *n* = 598 for the prenatal stage, and *n* = 517 for the postnatal stage. The prenatal stage included measurements of the blood lead levels during the second and third trimesters of pregnancy. The postnatal stage included measurements of the blood lead levels at birth and at 1, 2, and 4 years of age.

Supplementary Table S2 shows the results of the longitudinal association models between the BLLs at each stage (i.e., prenatal, postnatal, and all stages) and continuous IMetS between the 72- and 96-month study visits. We observed a statistically significant inverse association between children with HPb during all stages and TC levels (β = −5.40, 95%CI = −9.75 to −1.04). Similarly, HPb had an inverse association with SBP (β = −1.92, 95%CI = −3.72 to −0.11) at all stages. Additionally, we observed suggestive inverse associations between HPb and cLDL and BF%, which did not reach statistical significance, during the prenatal stage (β = −3.26, 95%CI = −7.03 to 0.51; β = −1.45, 95%CI = −2.95 to 0.05).

Table 5 shows the results of the regressions assessing the relationships with bone Pb measures as continuous variables. We observed that higher Pb levels in the patella were positively associated with cHDL levels <45 mg/dl (OR = 1.03, 95%CI = 1.00–1.07). We also observed an inverse association among children with higher tibia Pb levels and TGs: children with higher tibia Pb levels were 5% less likely to have elevated TGs (OR = 0.95, 95%CI = 0.91–0.99).

**Table 5:** Associations between the lead levels in the trabecular (patella) and cortical (tibia) bone and indicators of metabolic syndrome categorized according to the cutoff points.

	**Patella^a^**		**Tibia^a^**	
	**OR**	**95%CI**	**OR**	**95%CI**
Glucose, ≥100 mg/dl			1.02	0.97–1.07
HbA1C, ≥5.7%	0.99	0.95–1.03	0.99	0.94–1.03
Total cholesterol, >170 mg/dl	1.00	0.96–1.03	0.95	0.91–0.99
Triglycerides, >75 or >90 mg/dl	0.98	0.95–1.01	**0.95** ^c^	**0.91–0.99**
cHDL, <45 mg/dl	**1.03** ^c^	**1.00–1.07**	1.00	0.96–1.04
cLDL, >110 mg/dl	1.01	0.97–1.05	0.97	0.92–1.03
Body fat percentage, >80th percentile				
Waist circumference, >80th percentile	0.99	0.92–1.07	0.99	0.90–1.09
BMI for age, *z*-scores	0.93	0.85–1.01	0.98	0.89–1.09
Systolic blood pressure, >90th percentile	0.99	0.95–1.02	0.99	0.95–1.04
Diastolic blood pressure, >90th percentile	0.97	0.93–1.01	0.98	0.93–1.03

*cHDL, high-density lipoprotein cholesterol; cLDL, low-density lipoprotein cholesterol*.

a *Models adjusted for socioeconomic status, sex, maternal age, size for gestational age, infant age, and parity*.

*^b^Results in bold with statistically significant differences (p < 0.05)*.

c *Results in italics with marginally significant differences (p < 0.09)*.

The Pb data available decreased to 75.6% for stage 12 and to 64.3% for stage 24. To assess the effect of loss to follow-up, we assessed the associations with a subsample of children who had complete data for all visits. The results were similar to those reported in the initial sample. Our results did not change in the models using continuous BLLs (results not shown) or in the sensitivity analysis including children with complete data (Supplementary Table S3). Finally, the results for the sex-stratified models evaluating possible effect modifications showed no statistically significant differences in the association between boys and girls (Supplementary Table S4).

## Discussion

Our study found associations between Pb exposure in early life and the different early-stage risk indicators of MetS. Although in some cases the direction of such associations was opposite that of our hypotheses, these suggest a disruption of the expected normality and should be evaluated. Pre- and postnatal HPb exposures were inversely associated with children's TC, WC, BMI, elevated TGs, and blood pressure at ages 6–12 years. Pb in cortical bone was associated with higher odds of having cHDL <45 mg/dl, and Pb in trabecular bone showed lower odds of elevated TGs. Evidence from studies in children remains controversial, and our results are in line with some, but not all. To illustrate this, below, we present a summary of the associations reported in other studies; details can be found in Supplementary Table S5. In general, the differences between our study and others reported here could be due to the study design (many were cross-sectional), the Pb biomarkers used, differences in ethnicity and age, and the use of different cutoff points for IMetS. Contrary to most prior literature (20, 21, 26, 27), we observed no evidence of sex-specific associations. A similar null sex-specific association was found between BLLs and TC (20).

### Total Cholesterol

Our study suggests that HPb, both postnatally and during all stages, was associated with higher odds of having lower TC levels. This result could be explained by the effect of prenatal exposure to Pb in the regulation of cholesterol metabolism (20, 28). According to the study by Liu et al., exposure to Pb was inversely associated with the TC levels in boys (20), and the direction of the association was similar to what we have observed in the present study. Kupsco et al. found no association between the BLLs in pregnancy and TC in 4- to 6-year-old children (17). In the study by Poursafa et al. (21), positive associations were found across quartiles of the BLLs and TC in children and among girls.

### cHDL

In our study children with higher levels of Pb in the patella were more likely to have cHDL levels <45 mg/dl. These results were similar to prior evidence, which showed that prenatal BLLs were inversely associated with cHDL at age 10–18 years (20) although a null association was reported by Poursafa et al. (21) in Iranian pre- and adolescents.

### Triglycerides

Prenatal BLLs and tibia Pb levels were inversely associated with TGs. Most of the evidence has reported null associations (17, 20), yet one study on Iranian boys and girls found that an increase in the quartiles of BLLs was positively associated with TGs (21). In our study, concordance between the direction of the associations between BLLs in the prenatal stage and the cumulative Pb levels measured in the tibia may suggest that exposure during pregnancy could impact the TGs levels in 6- to 12-year-old children.

### BMI

Not surprisingly, we observed that an increased prevalence of overweight and obesity added to the increased WC measures and BF% at the stage 96 study visit, which could be due to changes in body composition associated with age (29).

Similar to what have been reported, we found an inverse association between HPb and BMI for age (30–34). This evidence shows that both prenatal (32, 34) and postnatal (33) exposures to Pb adversely impact children's growth. In the study by Shao et al., negative associations were found between urinary levels of Pb and overweight and obesity, being stronger among 6- to 12-year-olds compared to those in 13- to 19-year-olds (30). In the study by Liu et al., Pb levels in the patella were negatively associated with lower child BMI *z*-scores (β = −0.02, 95%CI = 0.03 to −0.01). In prior studies of the PROGRESS cohort, Renzetti et al. (Pb during the last two trimesters of pregnancy, delivery and postpartum, with BMI in children at 4–6 years) (32) and Kupsco et al. (BLLs during the third trimester of pregnancy and the BMI *z*-scores at 48 months) (17) reported null associations. Similarly, null associations were found by Afeiche et al. between longitudinal exposure to Pb and BMI in 48-month-old Mexican children. However, other findings showed that children with high exposure in all windows of development (prenatal, infancy, and early infancy) were almost 1 cm (β = −0.98, 95%CI = −1.86 to −0.10) shorter than children with low exposure during those periods, in birth cohorts in Mexico City (35).

Although the evidence is not conclusive, we propose three main biological mechanisms that could be involved. Firstly, the impact of Pb exposure *in utero* resulting in lower weight (36, 37) and height at birth (37): this effect can continue during early childhood (38). A second mechanism is the action of Pb as an endocrine disruptor by reducing the response of hormones such as insulin like-growth factor (39) and the action of cortisol that could affect the hypothalamus–pituitary–adrenal axis (40). The third and most reported mechanism involves the main effect of Pb on bone growth by impairing the function of bone cells (osteoblasts), the mineralization of bone, and the ability of the bone to respond to hormonal regulation, which can also affect the bone cartilage (41, 42).

### Waist Circumference and Body Fat Percentage

We found a similar association between HPb and WC to that reported in the study by Liu et al., where the Pb levels in the patella were inversely associated with WC (β = −0.12 cm, 95%CI = 0.22 to −0.03) and BF% (β = −0.09%, 95%CI = 0.17 to −0.01) (34). Similar associations were also reported by Deierlein et al. in a study of girls (33). In the case of BF%, null associations were found in our study, similar to results previously reported in the PROGRESS cohort with children in other developmental stages (17, 32).

### Systolic and Diastolic Blood Pressure

Contrary to what we expected, our results showed that HPb was associated with less likelihood to have elevated SBP and DBP (above the 90th percentile). Positive associations have been shown between Pb exposure and SBP using different biomarkers such as maternal toenail (27), maternal tibia bone (26), and children's BLLs (21). Null associations between maternal exposure to Pb and blood pressure were reported by Skröder et al. in a cohort of 4.5-year-olds rural in Bangladesh (43) and by Kupsco et al. in the same study population as ours, children 4–6 years old (17). Poursafa et al. showed positive associations between the quartiles of exposure to Pb and SBP and DBP. More studies are needed to explain the biological mechanisms that could produce these changes on vascular systems.

We are aware of the possibility of a type 1 error due to multiple comparisons; however, after Bonferroni adjustments, the presented associations between HPb and TC were still statistically significant. One of our study limitations is having missing BLLs in the 12- and 24-month postnatal stages, but the results in the models indicated that these losses did not influence the associations found.

Exposure to lead continues to be a public health problem in Mexico since the main source of exposure is the use of lead-glazed low-temperature ceramics to prepare, serve, and store food. Lead will leach into food with each use, even in very old and worn-out dishes. This type of ceramics is widespread in Mexico, and recent studies using representative national data have shown a clear association between their use and BLLs in 1- to 4-year-old children (44, 45).

The strengths of this study include it being the first epidemiological study that evaluated the association between repeated measures of exposure to Pb (separately during prenatal, postnatal, and cumulative early life stages) and the risk of IMetS longitudinally at two different time points, between ages 6 and 12 years. A particular strength is our extensive data on Pb exposure in both blood (acute exposure, with a half-life of 25 days) and bone [indicator of chronic exposure (half-life of decades) and endogenous exposure during pregnancy and long-term exposure in mothers]. The PROGRESS cohort is a prospective study that collected data on 11 risk factors of IMetS evaluated at two different ages, as well as covariates for model adjustment.

## Conclusion

In this study, a longitudinal comprehensive assessment of early risk indicators of MetS in children, we observed small changes according to the biomarkers and developmental windows of Pb exposure. These changes should be assessed across adolescence and early adulthood.

## Data Availability Statement

The raw data supporting the conclusions of this article will be made available by the authors, without undue reservation.

## Ethics Statement

The studies involving human participants were reviewed and approved by National Institute of Public Health, Mexico Icahn School of Medicine at Mount Sinai, New York, USA. Written informed consent to participate in this study was provided by the participants' legal guardian/next of kin.

## Author Contributions

KM-S and MT-O: conceptualization, methodology, formal analysis, writing—original draft, writing—review and editing, and visualization. AA and EO-P: conceptualization, methodology, formal analysis, writing—original draft, and writing—review and editing. MP-Z: investigation and writing—review and editing. AM-G: investigation. RW and MM: writing—review and editing, supervision, project administration, and funding acquisition. AS: methodology, writing—original draft, and writing—review and editing. All authors contributed to the article and approved the submitted version.

## Funding

This work was supported by funding from the National Institute of Environmental Health Sciences P30 ES023515, R01 ES014930, R01 ES013744, and R24 ES028522 to RW. This work is a product of KM-S Master's thesis at the National Institute of Public Health, Mexico—as such we have no funding for the publication fees of this manuscript. MT-O is a researcher at the Mexican Social Security Institute and does not have funding resources for publication.

## Conflict of Interest

The authors declare that the research was conducted in the absence of any commercial or financial relationships that could be construed as a potential conflict of interest.

## Publisher's Note

All claims expressed in this article are solely those of the authors and do not necessarily represent those of their affiliated organizations, or those of the publisher, the editors and the reviewers. Any product that may be evaluated in this article, or claim that may be made by its manufacturer, is not guaranteed or endorsed by the publisher.
