# Supplementary material for: Prenatal and Early Childhood Exposure to Lead and Repeated Measures of Metabolic Syndrome Risk Indicators From Childhood to Preadolescence
Source: Front Pediatr. 2021 Oct 29;9:750316. doi: 10.3389/fped.2021.750316 (PMC8586085; doi:10.3389/fped.2021.750316)
Supplement: Supplementary file 1 [file Data_Sheet_1.docx]

| **Supplementary Table 1. Descriptive characteristics of the Metabolic Syndrome Risk Indicators distributed according to percentiles and sex in the children of the PROGRESS Cohort.** | | | | | | | | | | | | |
| --- | --- | --- | --- | --- | --- | --- | --- | --- | --- | --- | --- | --- |
|  | Boys | | | | | | Girls | | | | | |
| Indicator | p25 | p50 | p75 | p80 | p90 | p99 | p25 | p50 | p75 | p80 | p90 | p99 |
| Waist Circumference (cm) | | | | | | | | | | | | |
| Stage 72 | 51.3 | 54.3 | 58.0 | 60.4 | 67.9 | 80.8 | 51.1 | 54.9 | 60.5 | 62.0 | 67.2 | 83.8 |
| Stage 96 | 59.6 | 65.5 | 73.8 | 76.0 | 83.7 | 98.2 | 59.1 | 65.2 | 74.1 | 76.0 | 82.2 | 98.5 |
| Body Fat Percentage (%) | | | | | | | | | | | | |
| Stage 72 | 18.3 | 22.6 | 27.9 | 29.6 | 34.1 | 45.6 | 20.3 | 24.4 | 30.8 | 32.5 | 37.5 | 48.3 |
| Stage 96 | 22.4 | 29.6 | 37.6 | 39.3 | 42.8 | 48.2 | 23.8 | 30.5 | 37.2 | 39.2 | 42.3 | 50.6 |
| Systolic Blood Pressure (mmHg) | | | | | | | | | | | | |
| Stage 72 | 93.5 | 102.0 | 113.5 | 116.5 | 124.0 | 139.0 | 91.5 | 100.5 | 109.7 | 112.5 | 119.5 | 135.0 |
| Stage 96 | 105.8 | 112.4 | 118.4 | 120.3 | 127.0 | 147.3 | 105.0 | 110.6 | 118.0 | 119.5 | 126.3 | 137.3 |
| Diastolic Blood Pressure (mmHg) | | | | | | | | | | | | |
| Stage 72 | 55.5 | 61.5 | 69.0 | 71.5 | 76.0 | 88.0 | 55.2 | 60.5 | 68.0 | 69.5 | 74.5 | 83.5 |
| Stage 96 | 66.0 | 70.7 | 75.7 | 77.0 | 79.7 | 93.7 | 65.2 | 69.5 | 74.5 | 75.3 | 79.3 | 90.0 |

| Supplementary Table 2. Association of higher lead exposure levels (above the median) and continuous risk indicators for metabolic syndrome. | | | | | | |
| --- | --- | --- | --- | --- | --- | --- |
|  | All Stages^1^ | | Prenatal^1^ | | Postnatal^1^ | |
|  | β | 95% CI | β | 95% CI | β | 95% CI |
| Glucose, mg/dL | 0.79 | -0.71,2.30 | -0.14 | -1.45,1.16 | -0.10 | -1.26,1.46 |
| HbA1C, % | -0.00 | -0.08,0.07 | 0.02 | -0.04,0.08 | -0.03 | -0.10,0.03 |
| Total Cholesterol, mg/dL | **-5.40** | **-9.75, -1.04** | *-3.29* | *-7.09,0.51* | -3.28 | -7.38,0.82 |
| Triglycerides, mg/dL | -3.95 | -10.9,3.02 | -4.51 | -10.5,1.55 | -0.90 | -7.62,5.81 |
| cHDL, mg/dL | -1.41 | -3.38,0.57 | -1.04 | -2.76,0.68 | -1.36 | -3.21,0.49 |
| cLDL, mg/dL | *-3.26* | *-7.03,0.51* | -1.44 | -4.73,1.85 | -1.77 | -5.31,1.78 |
| Fat body percentage, % | *-1.45* | *-2.95,0.05* | -0.61 | -1.92,0.70 | -0.67 | -2.11,0.77 |
| Waist Circumference, cm | -1.02 | -2.61,0.56 | -0.54 | -1.93,0.85 | -0.42 | -1.92,1.09 |
| BMI, kg/m^2^ | -0.45 | -0.99,0.09 | -0.19 | -0.67,0.28 | -0.15 | -0.67,0.36 |
| Systolic blood pressure, mmHg | **-1.92** | **-3.72, -0.11** | *-1.49* | *-3.07,0.08* | 0.10 | -1.57,1.77 |
| Diastolic blood pressure, mmHg | -1.07 | -2.42,0.28 | -0.83 | -2.01,0.35 | 0.39 | -0.88,1.66 |
| ^1Models adjusted by maternal characteristics: socioeconomic status, maternal age, and parity; characteristics of infants: sex, size for gestational age, and infant age.^  ^*Results with statistically significant differences (p <0.05)^  *^†Results with marginally significant differences (p <0.0^*  ^Glucose, TC, CHDL and CLDL n = 586 for all stages, n = 585 prenatal stage and n = 509 postnatal stage.^  ^Glycosylated hemoglobin and TGA n = 583 for all stages, n = 582 prenatal stage and n = 508 postnatal stage.^  ^WC and BMI n = 601 for all stages, n = 600 prenatal stage and n = 519 postnatal stage.^  ^Systolic and Diastolic blood pressure n = 599 for all stages, n = 598 prenatal stage and n = 517 postnatal stage.^  ^Prenatal stage includes measurements of blood lead levels during the 2nd and 3rd trimesters of pregnancy. Postnatal stage includes measurements of blood lead levels at birth, 1 year, 2 and 4 years of age.^ | | | | | | |

| Supplementary Table 3. Association of levels of exposure to Pb and indicators of metabolic syndrome in a subsample with complete data. | | | | | | |
| --- | --- | --- | --- | --- | --- | --- |
|  | All Stages^1^ | | Prenatal^1^ | | Postnatal^1^ | |
|  | β | 95% CI | β | 95% CI | β | 95% CI |
| Glucose, mg/dL |  |  |  |  |  |  |
| Above the median | *2.04* | *-0.21,4.30* | -0.06 | -2.12,1.99 | **2.33** | **0.31,4.36** |
| HbA1C, % |  |  |  |  |  |  |
| Above the median | **0.17** | **0.05,0.30** | **0.14** | **0.03,0.25** | 0.05 | -0.06,0.16 |
| Total Cholesterol, mg/dL |  |  |  |  |  |  |
| Above the median | -4.91 | -11.9,2.06 | -2.99 | -9.31,3.31 | -3.30 | -9.59, -2.98 |
| Triglycerides, mg/dL |  |  |  |  |  |  |
| Above the median | -7.30 | -19.7,5.09 | 0.58 | -10.6,11.8 | -0.49 | -11.7,10.7 |
| cHDL, mg/dL |  |  |  |  |  |  |
| Above the median | 0.06 | -3.01,3.15 | -1.01 | -3.79,1.76 | -1.72 | -4.48,1.04 |
| cLDL, mg/dL |  |  |  |  |  |  |
| Above the median | -3.51 | -9.57,2.54 | -2.09 | -7.57,3.37 | -1.48 | -6.94,3.97 |
| Body fat percentage, % |  |  |  |  |  |  |
| Above the median | -0.66 | -3.00,1.67 | 0.25 | -1.86,2.37 | -0.38 | -2.49,1.71 |
| Waist Circumference, cm |  |  |  |  |  |  |
| Above the median | -1.21 | -3.74,1.32 | -0.66 | -2.95,1.62 | -0.23 | -2.51,2.04 |
| BMI, kg/m^2^ |  |  |  |  |  |  |
| Above the median | -0.14 | -1.01,0.71 | -0.05 | -0.83,0.72 | 0.14 | -0.63,0.92 |
| Systolic blood pressure, mmHg |  |  |  |  |  |  |
| Above the median | **-4.13** | **-6.84,-1.42** | -1.88 | -4.36,0.59 | **-2.75** | **-5.21, -0.30** |
| Diastolic blood pressure, mmHg |  |  |  |  |  |  |
| Above the median | **-3.40** | **-5.54,-1.26** | -1.60 | -3.56,0.35 | **-2.27** | **-4.21,-0.32** |
| ^1Models adjusted by maternal characteristics: socioeconomic status, maternal age and parity; characteristics of infants: sex, size for gestational age, and infant age.^  ^*Results with statistically significant differences (p <0.05)^  *^†Results with marginally significant differences (p <0.0^*  ^For all indicators of metabolic syndrome n = 206 all stages, prenatal and postnatal^  ^Prenatal stage includes measurements of blood lead levels during the 2nd and 3rd trimesters of pregnancy. Postnatal stage includes measurements of blood lead levels at 4 years of age.^ | | | | | | |

| Supplementary Table 4. Association of Higher Lead Exposure Levels (above the median) and Indicators of Metabolic Syndrome by Sex. | | | | | |
| --- | --- | --- | --- | --- | --- |
|  | Boys^1^ | | Girls^1^ | |  |
|  | n | β (95% CI) | n | β (95% CI) | p-value |
| Glucose, mg/dL | 298 | 1.59(-0.63,3.83) | 288 | 0.30 (-1.71,2.31) | 0.547^a^ |
| HbA1C, % | 298 | 0.02(-0.09,0.14) | 288 | -0.01(-0.11,0.09) | 0.912^a^ |
| Total Cholesterol, mg/dL | 298 | -7.26(-13.7,-0.73) | 288 | -3.55(-9.33,2.23) | 0.303^a^ |
| Triglycerides, mg/dL | 298 | -4.85(-15.0,5.28) | 288 | -4.25(-13.8,5.32) | 0.939^a^ |
| cHDL, mg/dL | 298 | -1.25(-4.19,1.67) | 288 | -1.18(-3.86,1.49) | 0.876 ^a^ |
| cLDL, mg/dL | 298 | -5.10(-10.8,065) | 288 | -1.54(-6.45,3.37) | 0.284 ^a^ |
| Body fat percentage, % | 305 | -1.45(-3.58,0.68) | 289 | -1.33(-3.46,0.80) | 0.993 ^a^ |
| Waist Circumference, cm | 308 | -0.54(-2.77,1.68) | 293 | -1.35(-3.63,0.92) | 0.592 ^a^ |
| BMI, kg/m^2^ | 308 | -0.45(-1.22,031) | 293 | -0.45(-1.23,0.33) | 0.886 ^a^ |
| Systolic blood pressure, mmHg | 307 | -2.40(-5.00,0.18) | 292 | -1.19(-3.71,1.31) | 0.494 ^a^ |
| Diastolic blood pressure, mmHg | 307 | -1.64(-3.66,0.36) | 292 | -0.31(-2.13,1.51) | 0.286 ^a^ |
| ^1 Models adjusted by socioeconomic status, sex, maternal age, size for gestational age, infant age, and parity.^  ^a p value of the interaction between the exposure category and sex^  ^* Results with statistically significant differences (p <0.05)^  ^† Results with marginally significant differences (p <0.09).^  ^Models included data from all stages.^ | | | | | |

| **Supplementary Table 5. Previous studies published on the association between Lead exposure and indicators of metabolic syndrome in pediatric population.** | | | | | | | | |
| --- | --- | --- | --- | --- | --- | --- | --- | --- |
| ***IMetS*** | ***Direction of the association in this study*** | ***Author*** | ***Year*** | ***Study Population*** | ***Pb Biomarker*** | ***Study Design*** | ***Indicators of MetS*** | ***Direction of the association in comparison study*** |
| Glucose | Null | Liu, *et al*^20^ | 2020 | Mexican birth cohorts, ELEMENT, 369 children, 186 girls and 183 boys at ages 10 to 18 years. | BLLs at 1^st^ trimester of pregnancy classified into two categories: ≥5 µg/dL and ≤5 µg/dL | Longitudinal | Fasting Glucose | Null |
|  |  | Poursafa *et al*^21^ | 2014 | Iranian survey, CASPIAN-III study, 320 adolescents aged 10 to 18 years. 160 with MetS and 160 healthy controls. | BLLs in adolescents classified into quartiles | Cross-sectional | Fasting Glucose | Positive across quartiles of BLLs and Glucose |
| HbA1C | Null | Kupsco *et al* ^17^ | 2018 | Mexican birth cohort, PROGRESS, 548 mother- child pairs. Outcome measured at 4-6 years old | BLLs at 2^nd^ trimester of pregnancy | Longitudinal | HbA1C | Null |
| Total Cholesterol (TC) | Negative between HPb pre-postnatal and TC  Negative between HPb postnatal and TC | Liu, *et al*^20^ | 2020 | Mexican birth cohorts, ELEMENT, 369 children, 186 girls and 183 boys at ages 10 to 18 years. | BLLs at 1^st^ trimester of pregnancy classified into two categories: ≥5 µg/dL and ≤5 µg/dL | Longitudinal | TC | Negative between BLLs ≥5 µg/dL and TC  Negative between BLLs ≥5 µg/dL and TC in boys. |
|  |  | Poursafa *et al*^21^ | 2014 | Iranian survey, CASPIAN-III study, 320 adolescents aged 10 to 18 years. 160 with MetS and 160 healthy controls. | BLLs in adolescents classified into quartiles | Cross-sectional | TC | Positive across quartiles of BLLs and TC  Positive across quartiles of BLLs and TC among girls |
|  |  | Kupsco *et al* ^17^ | 2018 | Mexican birth cohort, PROGRESS, 548 mother- child pairs. Outcome measured at 4-6 years old | BLLs at 2^nd^ trimester of pregnancy | Longitudinal | Non-HDL cholesterol | Null |
| Triglycerides  (TGA) | Negative between HPb pre-postnatal and TGA  Negative between Tibia Pb levels and TGA | Kupsco *et al* ^17^ | 2018 | Mexican birth cohort, PROGRESS, 548 mother- child pairs. Outcome measured at 4-6 years old | BLLs at 2^nd^ trimester of pregnancy | Longitudinal | TGA | Null |
|  |  | Liu, *et al*^20^ | 2020 | Mexican birth cohorts, ELEMENT, 369 children, 186 girls and 183 boys at ages 10 to 18 years. | BLLs at 1^st^ trimester of pregnancy classified into two categories: ≥5 µg/dL and ≤5 µg/dL | Longitudinal | TGA | Null |
|  |  | Poursafa *et al*^21^ | 2014 | Iranian survey, CASPIAN-III study, 320 adolescents aged 10 to 18 years. 160 with MetS and 160 healthy controls. | BLLs in adolescents classified into quartiles | Cross-sectional | TGA | Positive across quartiles of BLLs and TGA  Positive across quartiles of BLLs and TGA among girls  Positive across quartiles of BLLs and TG among boys |
| cHDL | Negative between patella Pb levels and HDL-C | Liu, *et al*^20^ | 2020 | Mexican birth cohorts, ELEMENT, 369 children, 186 girls and 183 boys at ages 10 to 18 years. | BLLs at 1^st^ trimester of pregnancy classified into two categories: ≥5 µg/dL and ≤5 µg/dL | Longitudinal | HDL-C | Negative between BLLs ≥5 µg/dL and HDL-C in boys |
|  |  | Poursafa *et al*^21^ | 2014 | Iranian survey, CASPIAN-III study, 320 adolescents aged 10 to 18 years. 160 with MetS and 160 healthy controls. | BLLs in adolescents classified into quartiles | Cross-sectional | HDL-C | Null |
| cLDL | Null | Liu, *et al*^20^ | 2020 | Mexican birth cohorts, ELEMENT, 369 children, 186 girls and 183 boys at ages 10 to 18 years. | BLLs at 1^st^ trimester of pregnancy classified into two categories: ≥5 µg/dL and ≤5 µg/dL | Longitudinal | LDL-C | Negative between BLLs ≥5 µg/dL and LDL-C  Negative between BLLs ≥5 µg/dL and LDL-C in boys |
|  |  | Poursafa *et al*^21^ | 2014 | Iranian survey, CASPIAN-III study, 320 adolescents aged 10 to 18 years. 160 with MetS and 160 healthy controls. | BLLs in adolescents classified into quartiles | Cross-sectional | LDL-C | Null |
| BMI | Negative between HPb in all stages and BMI for age (BFA) | Renzetti *et al* ^32^ | 2017 | Mexican birth cohort, PROGRESS, 513 mother- child pairs. Outcome measured at 4-6 years old | BLLs at 2^nd^ and 3^rd^ trimester of pregnancy  BLLs maternal and umbilical cord.  Bone Pb levels tibia and patella | Longitudinal | BMI for age (BFA)  Other findings: Weight for age (WFA)  Height for age (HFA) | Null  Negative between 3rd BLLs and WFA  Negative between 3rd BLLs and HFA |
|  |  | Kupsco *et al* ^17^ | 2018 | Mexican birth cohort, PROGRESS, 548 mother- child pairs. Outcome measured at 4-6 years old | BLLs at 2^nd^ trimester of pregnancy | Longitudinal | BMI | Null |
|  |  | Liu *et al*^34^ | 2019 | Mexican birth cohorts, ELEMENT, 248 children, at ages 8 to 16 years. | Bone Pb levels in tibia and patella  BLLs from children at ages 1 to 4 years | Longitudinal | BFA | Negative between Patella Pb levels and BFA |
|  |  | Deierlein *et al*^33^ | 2019 | The Breast Cancer and Environment Research Program a prospective puberty cohort. 683 US Girls enrolled at ages 6-8 years and ≥3 follow-up visits. | BLLs in girls at age ≤10 years | Longitudinal | BFA  Other findings:  HFA | Negative between BLLs and BFA among girls  Negative between BLLs and HFA among girls |
|  |  | Shao *et al*^30^ | 2017 | Data from the National Health and Nutrition Examination Survey (1999–2011). 6602 US children aged 6–19 years were analyzed. | Urinary levels of Pb | Cross-sectional | BFA | Negative between BLLs and BFA among girls |
| Waist Circumference (WC) | Negative between HPb in all stages and WC | Liu *et al*^34^ | 2019 | Mexican birth cohorts, ELEMENT, 248 children, at ages 8 to 16 years. | Bone Pb levels in tibia and patella  BLLs from children at ages 1 to 4 years | Longitudinal | WC | Negative between Patella Pb levels and WC |
|  |  | Deierlein *et al*^33^ | 2019 | The Breast Cancer and Environment Research Program a prospective puberty cohort. 683 US Girls enrolled at ages 6-8 years and ≥3 follow-up visits. | BLLs in girls at age ≤10 years | Longitudinal | WC | Negative between BLLs and WC among girls |
| Body fat percentage (BF%) | Null | Renzetti *et al* ^32^ | 2017 | Mexican birth cohort, PROGRESS, 513 mother- child pairs. Outcome measured at 4-6 years old | BLLs at 2^nd^ and 3^rd^ trimester of pregnancy  BLLs maternal and umbilical cord.  Bone Pb levels tibia and patella | Longitudinal | PBF  Other findings:  Body fat mass | Null  Null |
|  |  | Kupsco *et al* ^17^ | 2018 | Mexican birth cohort, PROGRESS, 548 mother- child pairs. Outcome measured at 4-6 years old | BLLs at 2^nd^ trimester of pregnancy | Longitudinal | PBF | Null |
|  |  | Liu *et al*^34^ | 2019 | Mexican birth cohorts, ELEMENT, 248 children, at ages 8 to 16 years. | Bone Pb levels in tibia and patella  BLLs from children at ages 1 to 4 years | Longitudinal | PBF  Other findings:  Sum of skinfolds | Negative between Patella Pb levels and PBF  Negative between Patella Pb levels and Sum of skinfolds |
|  |  | Deierlein *et al*^33^ | 2019 | The Breast Cancer and Environment Research Program a prospective puberty cohort. 683 US Girls enrolled at ages 6-8 years and ≥3 follow-up visits. | BLLs in girls at age ≤10 years | Longitudinal | PBF | Negative between BLLs and PBF among girls |
| SBP | Negative between HPb pre-postnatal and SBP | Kupsco *et al* ^17^ | 2018 | Mexican birth cohort, PROGRESS, 548 mother- child pairs. Outcome measured at 4-6 years old | BLLs at 2^nd^ trimester of pregnancy | Longitudinal | SBP | Null |
|  |  | Poursafa *et al*^21^ | 2014 | Iranian survey, CASPIAN-III study, 320 adolescents aged 10 to 18 years. 160 with MetS and 160 healthy controls. | BLLs in adolescents classified into quartiles | Cross-sectional | SBP | Positive across quartiles of BLLs and SBP among girls  Positive across quartiles of BLLs and SBP among boys |
|  |  | Skröder *et al*^43^ | 2016 | MINIMat trial, a food and multi-micronutrient supplementation trial in pregnancy, in rural Bangladesh. 1574 children were analyzed at 4.5 years old | BLLs during early pregnancy (14 weeks) and late pregnancy (30 weeks) | Longitudinal | SBP  Other findings:  Kidney volume  Estimated glomerular filtration rate (eGFR)  Serum Cystatin C | Null  Negative between BLLs in late pregnancy and Kidney volume  Null  Null |
|  |  | Farzan *et al*^27^ | 2018 | New Hampshire Birth Cohort, 323 mother-child pairs. Outcome measured at 5 years old | Levels of Pb measured in maternal toenails at 28 weeks of pregnancy and 6 weeks postpartum | Longitudinal | SBP | Positive between maternal Pb levels in toenails and SBP. |
|  |  | Zhang *et al*^26^ | 2012 | Mexican birth cohorts, ELEMENT, 457 mother-child pairs, at ages 8 to 16 years. | Bone Pb levels in tibia and patella  BLLs from umbilical cord | Longitudinal | SBP | Positive between maternal tibia Pb levels and SBP among girls |
| DBP | Negative between HPb pre-postnatal and DBP  Negative between HPb prenatal and DBP | Kupsco *et al* ^17^ | 2018 | Mexican birth cohort, PROGRESS, 548 mother- child pairs. Outcome measured at 4-6 years old | BLLs at 2^nd^ trimester of pregnancy | Longitudinal | DBP | Null |
|  |  | Poursafa *et al*^21^ | 2014 | Iranian survey, CASPIAN-III study, 320 adolescents aged 10 to 18 years. 160 with MetS and 160 healthy controls. | BLLs in adolescents classified into quartiles | Cross-sectional | DBP | Positive across quartiles of BLLs and DBP  Positive across quartiles of BLLs and DBP among girls  Positive across quartiles of BLLs and DBP among boys |
|  |  | Skröder *et al*^43^ | 2016 | MINIMat trial, a food and multi-micronutrient supplementation trial in pregnancy, in rural Bangladesh. 1574 children were analyzed at 4.5 years old | BLLs during early pregnancy (14 weeks) and late pregnancy (30 weeks) | Longitudinal | DBP | Null |
|  |  | Farzan *et al*^27^ | 2018 | New Hampshire Birth Cohort, 323 mother-child pairs. Outcome measured at 5 years old | Levels of Pb measured in maternal toenails at 28 weeks of pregnancy and 6 weeks postpartum | Longitudinal | DBP | Null |
|  |  | Zhang *et al*^26^ | 2012 | Mexican birth cohorts, ELEMENT, 457 mother-child pairs, at ages 8 to 16 years. | Bone Pb levels in tibia and patella  BLLs from umbilical cord | Longitudinal | DBP | Positive between maternal tibia Pb levels and DBP among girls |
